# JA-Ile-Macrolactone 5b Induces Tea Plant (*Camellia sinensis*) Resistance to Both Herbivore *Ectropis obliqua* and Pathogen *Colletotrichum camelliae*

**DOI:** 10.3390/ijms21051828

**Published:** 2020-03-06

**Authors:** Songbo Lin, Yanan Dong, Xiwang Li, Yuxian Xing, Miaomiao Liu, Xiaoling Sun

**Affiliations:** 1 Tea Research Institute, Chinese Academy of Agricultural Sciences, Hangzhou 310008, China; linsongbo@tricaas.com (S.L.); 18843176083@163.com (Y.D.); lixiwang0392@tricaas.com (X.L.); 82101185063@caas.cn (Y.X.); L17693229659@163.com (M.L.); 2 Key Laboratory of Tea Biology and Resources Utilization, Ministry of Agriculture, Hangzhou 310008, China

**Keywords:** JA-Ile-macrolactone, induced resistance, *Camellia sinensis*, *Ectropis obliqua*, *Colletotrichum camelliae*

## Abstract

Jasmonates (JAs), the group of lipid-derived hormones, were found to control the defense responses in a myriad of plants. Meaningfully, the macrolactones of 12-hydroxy jasmonate isoleucine (12OH-JA-Ile) were reported to induce the defensive response of wild tobacco. However, little to nothing has been known about the elicitation effect of JA-Ile-macrolactones on woody plants to harmful organisms, let alone its underlying mechanisms. Here, we first optimized the synthetic routine using mild toxic reagent isobutyl chloroformate instead of ethyl chloroformate for conjugation, and we used acetonitrile (MeCN) instead of ethyl alcohol for the better dissolution of *p*-toluenesulfonic acid (*p*-TsOH) to gain JA-Ile-macrolactones. JA-Ile-macrolactone **5b**-treated tea plants significantly inhibited the larvae weight gain of *Ectropis obliqua* larvae and the lesions caused by *Colletotrichum camelliae*. Furthermore, the expression level of *CsOPR3* was significantly upregulated in **5b**-treated leaves. Meanwhile, **5b** reduced the accumulation of eriodictyol 7-O-glucuronide (EDG) in tea plants, which was confirmed to promote the growth rate of *E. obliqua* larvae by artificial diet assay. In conclusion, our study proved that the exogenous application of **5b** could induce the tea plant resistance both to herbivore *E. obliqua* and pathogen *C. camelliae*, and EDG was identified as one of the secondary metabolites that could influence the growth rate of *E. obliqua*, but it did not directly influence the infection of *C. camelliae* in vitro. Further research should be carried out to clarify the mechanism through which **5b** induces tea plant resistance to *C. camelliae*.

## 1. Introduction

During the hundreds of millions of years of evolution with herbivores and pathogens, plants have acquired sophisticated strategies to defense against them [1], such as producing direct toxic and anti-nutritional compounds to hinder insect growth or pathogen infection, or emitting volatiles to attract natural enemies of herbivorous pests [2]. Accumulated lines have revealed that these plant defense responses are often initiated upon recognizing herbivore/pathogen-derived cues or chemical regulators, followed by the activation of defense-related phytohormone signaling pathways, such as jasmonic acid (JA), salicylic acid (SA), ethylene (ET), etc., to mediate specific defense responses [3]. Therefore, more evidence demonstrated that the exogenous application of phytohormones could elicit plant resistance against herbivores or pathogens either in herbs or in woody plants [4]. In recent years, the analogues of these phytohormones modulating the corresponding pathways have been investigated by researchers [5,6]. Several chemical elicitors have been commercially used in greenhouse disease control, such as benzo (1,2,3)-thiadiazole-7-carbothioic acid S-methyl ester (BTH), 2,6-dichloroisonicotinic acid (INA), β-aminobutyric acid (BABA), tiadinil (TDL), and pyraclostrobin [7,8,9,10,11]. Numerous chemical elicitors have been found to trigger direct or indirect plant-induced defense responses against herbivores in the laboratory, such as methyl jasmonate (MeJA), coronalon, laminarin, and so on [12,13,14,15]. Moreover, 2, 4-dichlorophenoxyacetic acid [16], JA, and cis-jasmone [17] have been verified to elevate the plant defense capability against herbivores in the field, which is an attractive prospect for pest controlling [18,19]. Unfortunately, no any chemical elicitor has been used commercially in pest control until now.

Jasmonates (JAs), a large family of lipid-derived plant hormones, play a dominant role in modulating the plant response to stresses [20,21,22,23]. Biotic attack or mechanical injury induce the synthesis and accumulation of JA [20]. After the perception of (+)-7-iso-Jasmonoyl-L-isoleucine (JA-Ile) by receptor CORONATINE INSENSITIVE 1 (COI1), transcriptional repressor JASMONATE ZIM-DOMAIN (JAZ) proteins would be degraded [24]. Then, the expression of genes, all the major classes of secondary metabolites, and proteins related with immunity and defense are induced. The exogenous application of JAs could induce the morphological and physiological responses of plants [25]. For instance, MeJA enhanced the resistance of *Arabidopsis thaliana* to both cabbage looper *Trichoplusia ni* and pathogen *Pseudomonas syringae pv tomato* [26,27]. Moreover, other JAs with biological activity including a lot of structural analogues of JA-Ile from nature and chemical synthesis have been reported continuously [28,29,30,31,32,33]. The conjugates of JA with L-leucine and L-valine that occur in nature have been found to upregulate the transcript accumulation of JA-responsive genes, but still a series of the synthetic modification of JA had not exhibited biological activity in the modulation of plant defenses [29]. The phytotoxin coronatine, a structural mimic of JA-Ile produced by the plant pathogen *P. syringae* [30], is considerably more active than JA-Ile in promoting the interaction of the COI1 receptor with the JAZ repressors in vitro [24]. Moreover, the two products of lactonization of 12-OH-JA-Ile were found to induce the defensive responses of wild tobacco *Nicotiana tabacum* against *Manduca sexta* [32,33]. Nevertheless, little to nothing has been known about the elicitation effect of JA-Ile-macrolactones on woody plants to harmful organisms, let alone its underlying mechanisms.

Chemical pesticides are still an efficient approach to control pests and pathogens in the agriculture, which have caused serious “3R” problems (resistance, resurgence, and residence) [34]. The tea plant, *Camellia sinensis*, is one of the most important woody-plantation crops in the world. Developing tea shoots and leaves are the raw material for commercial tea, while they often suffer damages from numerous harmful organisms, such as the tea geometrid (TG) *Ectropis obliqua*, one of the main leaf-feeding pests of tea plantation in China, and *Colletotrichum camelliae*, one of the dominant leaf diseases that usually causes anthracnose disease leading to plant wilt, shoot death, and fragility [35,36,37]. Previous results showed that TG caterpillars developed more slowly on JA-treated tea plants than on control plants, and the larvae weight gain depended on the JA dosage and their infestation time [38]. Furthermore, a series of genes of the JA signaling pathway such as 12-oxophytodienoate reductase 3 (*CsOPR3*), hydroperoxide lyase (*CsHPL*), Acyl-CoA oxidase (*CsACX1, 3*), polyphenol oxidases (*CsPPO1, 2*), and lipoxygenase (*CsLOX*) were found to be upregulated by the infestation of TG [14,39,40,41,42]. Further investigation verified that the JA pathway plays a central role in tea plant defense against TG [43]. JA-Ile-macrolactones are the modification of JA-Ile; whether they could elicit tea plant resistance against harmful organisms or not needs to be verified.

In the present study, the synthetic route of the two macrolactones was optimized based on the previous reports firstly [33,44]. Secondly, the effects of macrolactones on the larvae weight gain of *E. obliqua* and the lesions caused by *C. camelliae* were measured. Thirdly, the potential mechanisms were explored preliminarily through the comparison of transcription expression levels of *CsOPR3* and the contents of 12 potential defensive flavonoids between control leaves and macrolactones-treated leaves. Finally, the effects of differential substance(s) on the larvae weight gain of TG caterpillars and the infection of *C. camelliae* were investigated.

## 2. Results

### 2.1. Optimized Synthesis of JA-Ile-Macrolactones

**Scheme 1:**
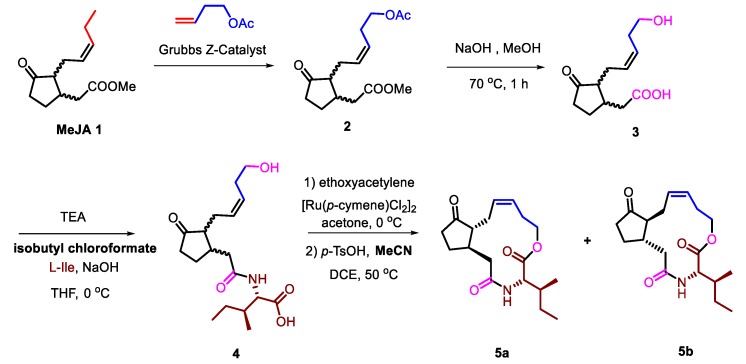
Optimized synthetic route of Jasmonoyl-L-isoleucine (JA-Ile)-macrolactones from Jimenez-Aleman et al. [33].

The synthesis began with the commercially available MeJA. 12-OH-jasmonic acid **3** was obtined through olefin metathesis catalyzed by Grubbs Z-Catalyst and saponification. Then, **3** was conjugated to L-isoleucine under alkaline condition. In this step, isobutyl chloroformate was used to replace hypertoxic ethyl chloroformate, which had been used in the reported article [33]. **4** was macrolactonized after the activation of carboxyl under acidic condition, in which *p*-toluenesulfonic acid (*p*-TsOH) was solved into acetonitrile (MeCN) to gain **5a** and **5b** (Scheme 1).

### 2.2. Effect of JA-Ile-Macrolactones on TG Performance

**Figure 1:**
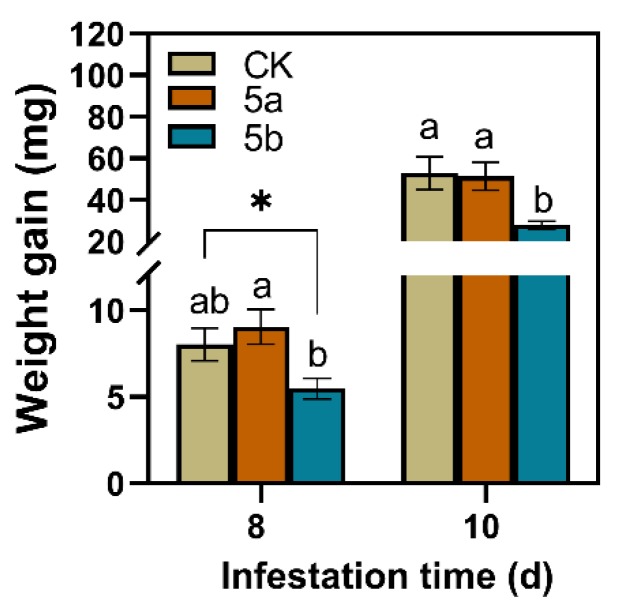
Mean larvae weight gain of tea geometrid (TG) (±SE, *n* = 22-28) fed on JA-Ile-macrolactones-treated tea leaves. Different letters indicate significant differences among treatments (*p* < 0.05, Turkey’s test). The asterisk indicates significant difference between treatment and control (*p* < 0.05, Student’s *t*-test).

Compared with the control, JA-Ile-macrolactone **5b**-treated leaves significantly reduced the larvae weight gain of TG (Figure 1). On the 8th and 10th days after the start of feeding, the weight gain of larvae that were fed on leaves treated with **5b** was significantly less than those fed on controls by 32% and 47%, respectively. However, there was no significant difference between the weight gain of the larvae fed on **5a**-treated tea leaves and those fed on the control leaves.

### 2.3. Effect of JA-Ile-Macrolactones on the Infection of *Colletotrichum camelliae*

**Figure 2:**
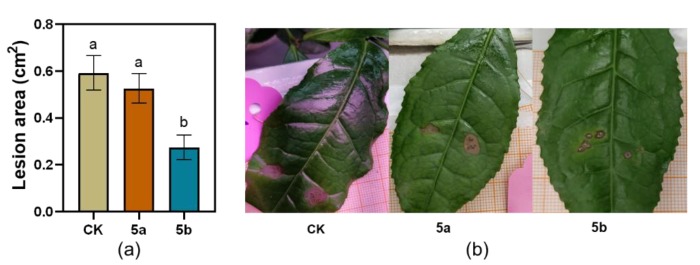
Effect of JA-Ile-macrolactones on the infection of *Colletotrichum camelliae*. (**a**) Mean lesion area of *C. camelliae* on tea plants (±SE, *n* = 12), different letters indicate significant differences among treatments (*p* < 0.05, Turkey’s test); (**b**) Photos of the infections.

Consistent with the effect of JA-Ile-macrolactones on TG larvae weight gain, **5b**-treated tea leaves inhibited the infected area of *C. camelliae* in 31% compared with that in control leaves on the 4th day after the start of inoculation (Figure 2).

### 2.4. Effects of JA-Ile-Macrolactones on Transcriptional Expression Level of CsOPR3

**Figure 3:**
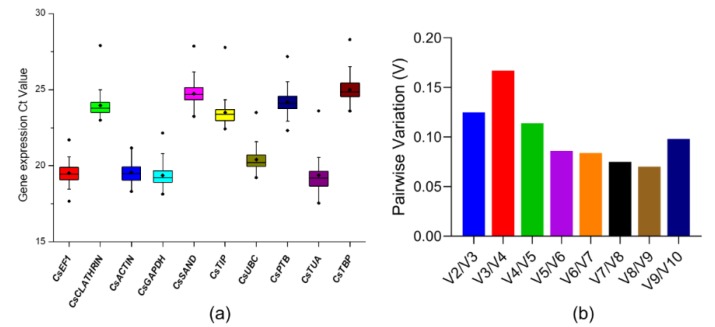
The selection and validation of endogenous reference genes. (**a**) Expression profiles of 10 candidate reference genes (RGs) in *C. sinensis*. The Ct mean value refers to the short bar; the box represents the 25th–75th percentiles, and the dark spots refer to extreme outliers; (**b**) Optimal number of reference genes for the normalization of *C. sinensis* under JA-Ile-macrolactones treatments. The pairwise variation (Vn/n + 1) was calculated by geNorm software to determine the optimal number of RGs required in the analysis of qPCR. Another RG will not significantly improve normalization when values are less than 0.15.

For the accurate qRT-PCR analysis in JA-Ile-Macrolactones plus lanolin treatment, we selected and validated endogenous reference genes (RGs) at first. Ten relatively stable RGs, *CsEF1*, *CsCLATHRIN*, *CsACTIN*, *CsGADPH*, *CsSAND*, *CsTIP*, *CsUBC*, *CsPTB*, *CsTUA* and *CsTBP*, which are frequently used as stable RGs in mRNA expression analysis of tea plants [45,46,47,48], were selected as the candidates to identify the best internal RG or combination. The Ct value of these RGs under JA-Ile-macrolactones treatments were recorded for evaluating the expression of 10 RGs. As shown in Figure 3a, the lowest mean Ct value was *CsGADPH* (19.37) and the highest mean Ct value was *CsTBP* (25.00). Then, the geNorm program [49] was used to calculate the pairwise variations between two sequential normalization factors (NFn and NFn + 1) to determine the optimal number of RGs for accurate normalization. As shown in Figure 3b, the pairwise variation at the V2/3 value was below 0.15, indicating that two RGs were enough for accurate normalization.

**Figure 4:**
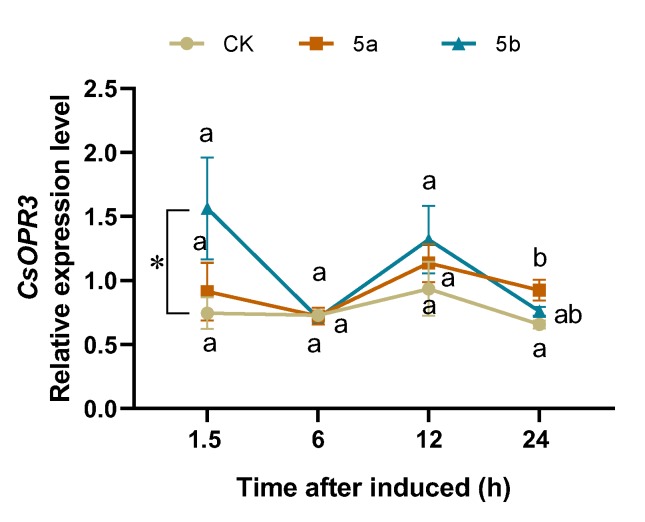
Mean relative expression level (±SE, *n* = 6) of *CsOPR3* in tea leaves elicited by JA-Ile-macrolactones. Different letters indicate significant differences among treatments (*p* < 0.05, Turkey’s test). The asterisk indicates significant difference between treatment and control (*p* < 0.05, Student’s *t*-test).

Based on the calculation of the four different computation programs geNorm [49], Normfinder [50], BestKeeper [51], and comparative ∆C_T_ (Table 1), *CsTBP* and *CsCLATHRIN* were chosen as the best combination of RGs. Normalizing with the combination, the relative expression level of *CsOPR3* in JA-Ile-macrolactones-treated tea leaves was significantly higher than that in the control, with 2.1-fold at 1.5 h (Figure 4).

### 2.5. The Elicitation of Flavonoids

**Figure 5:**
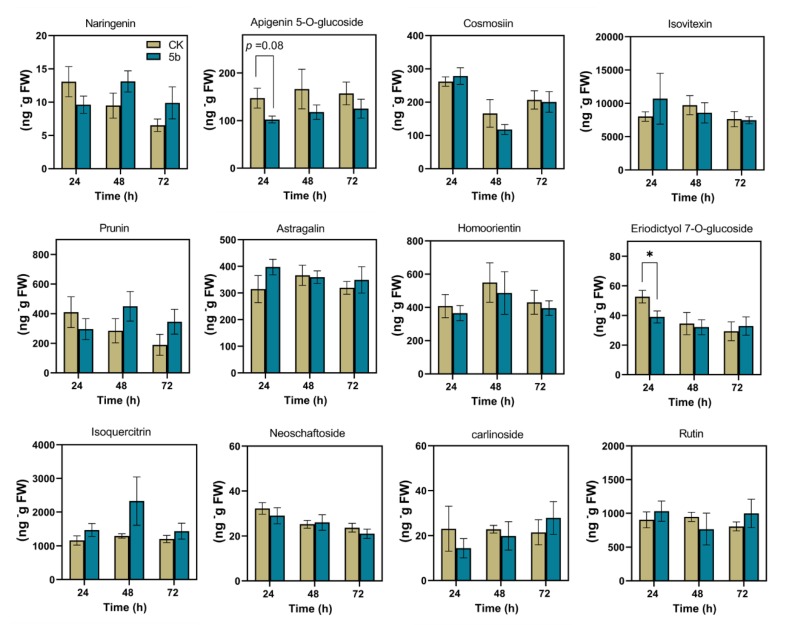
Mean content (±SE, *n* = 5) of 12 flavonoids elicited by JA-Ile-macrolactones. The asterisks indicate a significant difference between treatment and control (*p* < 0.05, Student’s *t*-test).

The contents of 12 flavonoids in plants were detected (Figure 5). The accumulations of most of these metabolites were influenced by **5b**, although there were no significant differences compared with the control. At 24 h, **5b** significantly inhibited the accumulation of eriodictyol 7-O-glucuronide (EDG) in 26% comparing with that in control.

### 2.6. Effects of EDG on the Performance of TG and the Infection of *Colletotrichum camelliae*

**Figure 6:**
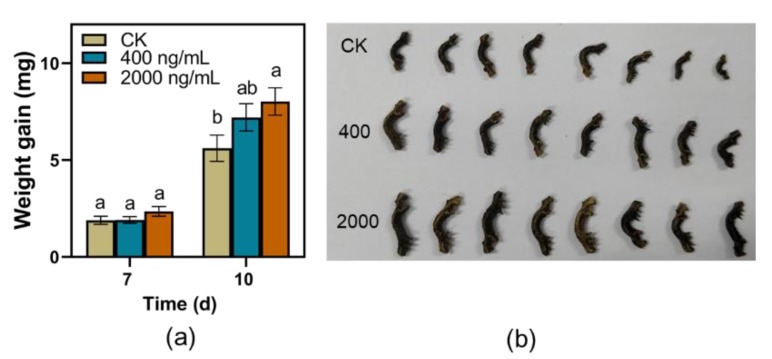
Effect of eriodictyol 7-O-glucuronide (EDG) on the performance of TG. (**a**) Mean larvae weight gain of TG (±SE, *n* = 25) fed on artificial diet supplemented with EDG in different concentrations; different letters indicate significant differences among treatments (*p* < 0.05, Turkey’s test); (**b**) Photos of their performance.

**Figure 7:**
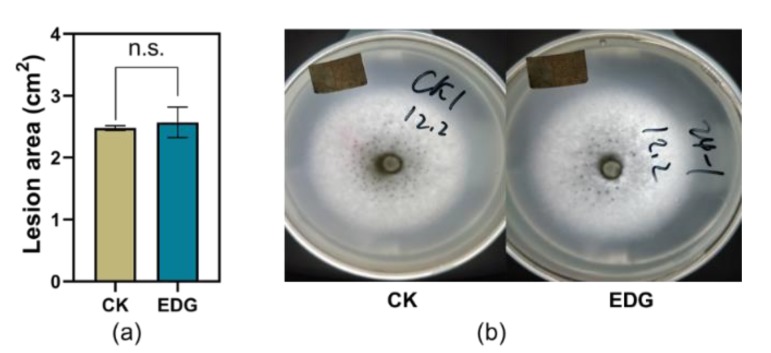
Effect of EDG on the performance of *C. camelliae.* (**a**) Mean infection area of *C. camelliae* on potato dextrose agar medium supplemented with EDG (±SE, *n* = 5); n.s. indicates no significant difference between treatment and control (*p* > 0.05, Student’s *t*-test); (**b**) photos of the infections.

The larvae weight gains of TG fed on artificial diet supplemented with EDG at a concentration of 2000 ng/mL were significantly higher than those fed on control after 10 d feeding in the current study (Figure 6a,b). However, potato dextrose agar medium supplemented with EDG did not affect the infection area of *C. camelliae* during the testing period (Figure 7a,b).

## 3. Discussion

Taking advantage of the plant-induced resistance against harmful organisms could be a promising alternative strategy for pests and pathogens management in the field [5,6]. A chemical elicitor is one of the most powerful tools in this domain [9,52,53,54]. Previously, several small molecule compounds have been reported to elicit tea plant resistance against herbivorous pests [14,38,55]. For example, (Z)-3-hexenol-treated [55] and JA-treated [38] tea plants inhibited the growth rate of TG larvae comparing to that of the control plants in laboratory; the β-1,3-glucan laminarin reduced the field population densities of tea green leafhopper (TLH) *Empoasca (Matsumurasca) onukii* Matsuda in the tea plantation [14]. Meanwhile, an interesting investigation reported that tobaccos treated with both JA-Ile-macrolactones **5a** and **5b** decreased the larvae mass gain and survival rate of *M. sexta* larvae [33]. In the present study, we firstly optimized the synthetic routine using mild toxic reagent isobutyl chloroformate instead of ethyl chloroformate for conjugation and MeCN instead of ethyl alcohol for the better dissolution of *p*-TsOH to gain JA-Ile-macrolactones **5a** and **5b** (Scheme 1), and then we investigated their induced resistance effect on tea plants. As shown in the results of this study (Figure 1), the TG larvae fed on **5b**-treated tea leaves grew significantly more slowly than that on control leaves, while no significant difference of TG larvae weight gain was observed between **5a**-treated leaves and controls. In addition, **5b**-treated tea leaves inhibited the lesions caused by *C. camelliae* in 31% compared with that in control leaves (Figure 2), which meant there was an induction of defense response of tea plants that repressed the infection of *C. camelliae*.

*OPR3* is a key member of genes encoding 12-oxophytodienoic acid reductases (OPRs), which is one of the key synthetase genes in the biosynthesis of JA in plant [56]. The upregulation of *OPR3* is also alongside the JA accumulation and the activation of the JA signal pathway [57]. The previous results of our lab showed that *CsOPR3* transcription could also be upregulated by the exogenous application of JA and TG infestation, which was alongside the accumulation of JA in tea plants [39]. In *A. thaliana*, the expression of *AtOPR3* could be induced by JA treatment and herbivore attack [58]. Given that **5b** is one of the JA derivatives, we select *CsOPR3* as the target gene to investigate the induction effect. As shown in the present study, **5b** significantly increased the expression level of *CsOPR3* in a tea plant at 1.5 h. However, due to the upregulation being at a relatively low level, it is not enough to represent that **5b** could elicit the JA signaling pathway of tea plants (Figure 4). This is the first report that JA-Ile-macrolactone **5b** could enhance the transcript accumulation of genes in plants. RNA-Seq analysis would be used in the following study to clarify how **5b** influences the signal pathways.

Plants orchestrate appropriate defense metabolites or proteins after signaling pathways were activated. Flavonoids, nicotine, anthocyanins, benzophenanthridine alkaloids, polyphenol oxidases (PPOs), and other secondary metabolites have been reported to act as defensive components, while the content of them often influence the plants’ resistance against herbivores and pathogens [23,59]. For example, nicotine is a toxic compound stimulated by JAs in tobacco plants, which was found to inhibit the growth rate of *M. sexta* [60]. Other evidence showed that an artificial diet supplemented with (+)-catechin, (–)-epicatechin, epicatechin, quercetin, rhamnetin, quercetin, or rutin inhibited the larvae growth rate of the European corn borer (*Pyrausta nubilalis* (*Hbn.*)) [61]. In tea plants, caffeine was found to be one of the metabolites against anthracnose (*Colletotrichum fructicola*) [62]. Furthermore, the theaflavin formation from catechins is induced by tea green leafhopper attacks, while the L-theanine content is increased by tea geometrid attacks [63]. Although EDG is one of the flavonoids originally isolated from the leaves of *Chrysanthemum morifolium cv.*, it had been reported to enhance the growth rate of cabbage looper (*Trichoplusia ni*) at the concentration of 10,000 ng/mL [64]. In the current study, the content of EDG in tea leaves was significantly repressed by the exogenous application of **5b** (Figure 5), while the artificial diet supplemented with EDG at concentration of 2000 ng/mL significantly increased the larvae weight gain of TG (Figure 6). JA-Ile-macrolactone **5b** treatment inhibited the weight gain of *E. obliqua* larvae through repressing the accumulation of EDG in tea leaves; thus, it is reasonable to infer that EDG probably is one of the stone components in tea plants. Not surprisingly, potato dextrose agar medium supplemented with EDG did not affect the lesions caused by *C. camelliae* in the testing concentration (Figure 7). Thus, other metabolites, not limited to the detected 12 flavonoids, or different concentrations of EDG, were supposed to be responsible for the inhibition of the infected of *C. camelliae*. Thus, although the effect of EDG on the performance of TG larvae has been evaluated indirectly, the mechanisms still need to be deeply investigated by using transcriptome and metabolome approaches.

In conclusion, the results in the present study revealed that the JA-Ile-macrolactone **5b** induces the tea plant resistance to TG and *C. camelliae.* EDG was identified as one of the secondary metabolites that could influence the growth rate of TG, but it did not directly influence the infection of *C. camelliae* in vitro. Further research should be carried out to deeply elucidate the underling mechanisms.

## 4. Materials and Methods

### 4.1. Insects

*E. obliqua* larvae were originally captured from tea fields in Hangzhou, Zhejiang, China, and raised in the net cages (75 × 75 × 75 cm^3^) with potted fresh tea shoots and kept in a controlled climate room programmed at 26 ± 2 °C, 70 ± 5% RH (relative humidity), and 12 h photo phase. After one generation, *E. obliqua* eggs were collected and hatched in the same condition; then, the 3-day-old larvae were used for the experiments.

### 4.2. The Optimized Synthesis of JA-Ile-Macrolactones

All reagents were purchased from commercial sources (Aladdin Biochemical Technology Co., Ltd., Shanghai, China) and used without further purification. ^1^H and ^13^C NMR spectra were recorded at 400 MHz on Bruker spectrometer. The synthesis of JA-Ile-macrolactones was optimized based on previously described reports [33]. In the synthesis of **2**, nitrogen flow was used to replace the dynamic vacuum. Hypertoxic ethyl chloroformate was changed to isobutyl chloroformate for the conjugation of **3** and L-Ile. MeCN was used to dissolve *p*-TsOH instead of ethyl alcohol. The NMR spectra data of all compounds were consistent with the previous report (Supplementary methods and Figure S2) [44].

### 4.3. Tea Plants and Treatments

Three-year-old Longjing 43 tea plants were used for the experiments, which were planted individually in plastic pots and grown in a greenhouse (26 ± 2 °C, relative humidity of 70–80%, 12 h photophase), irrigated once every other day, and fertilized with organic fertilizer once a month.

Then, 3.9 mg (12 μmol) of JA-Ile-macrolactones were dissolved into 120 μL of lanolin. The second leaf of each tea plant was smeared with 8 μL of lanolin supplemented with JA-Ile-macrolactones (0.8 µmol of each compound per leaf) and an equal amount of pure lanolin (control), respectively, and the treatments were repeated twice, 24 h apart. The treated leaves were harvested at 1.5 h, 6 h, 12 h, and 24 h after the second round of treatment. The experiment was carried out in a control climate room with the same conditions of cultivation. Each treatment replicated 6 times.

### 4.4. Effect of JA-Ile-Macrolactones-Treated Tea Leaves on E. obliqua Performance

Twenty-four hours after the second round of treatment, the treated leaf was covered with a fine-mesh sleeve, into which two 2nd instar *E. obliqua* larvae that had been starved for 6 h were introduced. After 8 days and 10 days of feeding, the larvae were weighed. During the testing process, the larvae would be transferred to another leaf of the same treatment sooner if it was almost exhausted. The experiment was replicated 22–28 times for each treatment.

### 4.5. Effect of JA-Ile-Macrolactones-Treated Tea Leaves on *C. camelliae* Infection

*C. camelliae* was isolated from diseased tea plants in Zhejiang (N 30°10’, E 120°5’), China and cultured on potato dextrose agar at 25 °C under 12 h photophase for 7 days. Twenty-four hours after the second round of treatment, the leaves were damaged with a sterile needle 5 times, and fungus cake (ID = 6 mm) was applied to the damaged part immediately (*n* = 12). After inoculation, the potted tea plants were maintained in the greenhouse, in which conditions were 26 ± 2 °C, 90 ± 5% RH (relative humidity), and 12 h photophase. Four days later, photos were taken of the infected leaves with a measuring scale, and the infection areas were calculated by adobe photoshop (Adobe Systems, San Jose, CA, USA). Twelve replications were carried out.

### 4.6. RNA Extraction, cDNA Synthesis, and Real-Time PCR (qPCR) Analysis

The total RNA of tea leaves was extracted via a TRIzol™ kit according to the protocol (TIANGEN, Beijing, China). Quality and concentration were examined by agarose-gel electrophoresis and spectrophotometer analysis. PrimerScript^®^ RT Reagent Kit (Takara, Dalian, China) was used to synthesize first-strand cDNA from the total RNA following the manufacturer’s protocol. The synthesized cDNAs were stored at −20 °C for further use after reverse transcription.

The primers of all the reference genes and *CsOPR3* are listed in Supplementary Table S1 according to the previous reports. The Real-Time PCR was performed on a LightCycler 480 (Roche Diagnostics, Mannheim, Germany) following the method as described by Huang [42]. The qPCR program included a preliminary step at 95 °C for 10 min, 45 cycles of a denaturation at 94 °C for 10 s, and an annealing step at 58 °C for 15 s and an extension at 72 °C for 12 s. The relative expressions were calculated by 2^−∆∆Ct^ method.

### 4.7. Flavonoids Quantification

The tea leaf powders (200 mg) were extracted with 1.0 mL of 80% methanol by vortexing (2 min) and ultrasonic extraction (15 min). After centrifugation (12,000 rpm, 30 min), 500 μL of supernatant was filtered through a 0.22 μm nylon filter membrane and measured by the UPLC/MS/MS. A Waters ACQUITY UPLC H-class system (Milford, MA, USA) coupled to a Waters Xevo TQ-S Micro triple-quadrupole mass spectrometer was used for UPLC/MS/MS analysis. A Waters ACQUITY UPLC HSS T_3_ analytical column (1.8 μm, 2.1 mm × 100 mm) was used at a temperature of 40 °C. The sample injection volume was 5 μL. The mobile phase consisted of 0.1% (*v*/*v*) formic acid in water (A), 0.1% (*v*/*v*) formic acid in acetonitrile (B). Elution was performed in gradient mode (0 min, 95% A, and 5% B at a flow rate of 0.25 mL·min^−1^; 0.5 min, 85% A, and 15% B at a flow rate of 0.25 mL·min^−1^; 5 min, 75% A, and 25% B at a flow rate of 0.25 mL·min^−1^; 9 min, 35% A, and 65% at a flow rate of 0.25 mL·min^−1^; 9.8 min, 0% A, and 100% B at a flow rate of 0.25 mL·min^−1^; 10.8 min, 0% A, and 100% at a flow rate of 0.40 mL·min^−1^; and 11 min, 95% A, and 5% B at a flow rate of 0.40 mL·min^−1^). The MS/MS analysis was conducted with electrospray ionization (ESI) in positive ion mode. All the flavonoids were detected under multiple reaction monitoring (MRM) mode. The optimized MRM transitions and retention times of 12 flavonoids are shown in the Supplementary Table S2. The contents of 12 flavonoids were absolutely quantified using the calibration curves of corresponding standard solutions (Supplementary Figure S1).

### 4.8. In Vitro Bioassay of E. obliqua Performance and *C. camelliae* Infection

As the method proposed by Yang et al. [38] and Wang et al. [62], EDG were added to the artificial diets or potato dextrose agar medium for the in vitro bioassay. Artificial diets supplemented with 0 (CK), 400, and 2000 ng/mL of EDG were used for antiherbivore bioassay. Each concentration replicated 25 times. Potato dextrose agar mediums supplemented with 0 (CK) and 2000 ng/mL of EDG were used for antifungal bioassay for 5 replications.

### 4.9. Statistical Analysis

All statistical analyses were performed by using the Statistica (Institute Inc., Cary, NC, USA). Differences of TG larvae weight gain, infection area of *C. camelliae*, and qPCR analysis of *CsOPR3* among treatments were analyzed via one-way ANOVA. If the ANOVA analysis was significant (*p* < 0.05), Tukey’s test for multiple comparisons was used to detect differences between groups. Student’s *t*-test was used for comparing difference between **5b** treatment and control. The stability of the candidate RGs was evaluated by BestKeeper, geNorm, NormFinder, the ΔCt method, and RefFinder.

## Figures and Tables

**Table 1:** Ranking of the reference genes expression under JA-Ile-macrolactones treatment.

Rank	geNorm		NormFinder		BestKeeper			ΔCt		Total
	RG ^1^	Stability	RG ^1^	Stability	RG ^1^	SD ^2^	r	RG ^1^	SD ^2^	
1	*CsCLATHRIN*	0.374	*CsTBP*	0.242	*CsEF1*	0.48	0.726	*CsTBP*	0.60	*CsCLATHRIN*
2	*CsUBC*	0.374	*CsCLATHRIN*	0.414	*CsACTIN*	0.49	0.615	*CsCLATHRIN*	0.66	*CsTBP*
3	*CsTBP*	0.399	*CsUBC*	0.430	*CsGAPDH*	0.49	0.734	*CsUBC*	0.67	*CsUBC*
4	*CsGAPDH*	0.542	*CsPTB*	0.479	*CsSAND*	0.49	0.742	*CsGAPDH*	0.71	*CsEF1*
5	*CsSAND*	0.580	*CsEF1*	0.489	*CsCLATHRIN*	0.51	0.874	*CsSAND*	0.71	*CsGAPDH*
6	*CsTIP*	0.595	*CsGAPDH*	0.490	*CsUBC*	0.53	0.824	*CsEF1*	0.71	*CsSAND*
7	*CsEF1*	0.618	*CsSAND*	0.492	*CsTBP*	0.53	0.936	*CsPTB*	0.72	*CsACTIN*
8	*CsPTB*	0.638	*CsTIP*	0.570	*CsTIP*	0.55	0.778	*CsTIP*	0.76	*CsPTB*
9	*CsACTIN*	0.657	*CsACTIN*	0.573	*CsPTB*	0.56	0.800	*CsACTIN*	0.77	*CsTIP*
10	*CsTUA*	0.734	*CsTUA*	0.940	*CsTUA*	0.76	0.675	*CsTUA*	1.04	*CsTUA*

^1^ Reference gene; ^2^ Standard deviation.

## Supplementary Materials

Supplementary Materials can be found at https://www.mdpi.com/1422-0067/21/5/1828/s1.

## Abbreviations

JA	Jasmonic acid
SA	Salicylic acid
ET	Ethylene
BTH	Benzo (1,2,3) thiadiazole-7-carbothioic acid S-methyl ester
INA	2,6-Dichloroisonicotinic acid
BABA	β-aminobutyric acid
TDL	Tiadinil
MeJA	Methyl jasmonate
JAs	Jasmonates
JA-Ile	(+)-7-iso-Jasmonoyl-L-isoleucine
COI1	CORONATINE INSENSITIVE 1
JAZ	JASMONATE ZIM-DOMAIN
TG	Tea geometrid
OPR	12-Oxophytodienoate reductase
HPL	hydroperoxide lyase
ACX	Acyl-CoA oxidase
PPO	polyphenol oxidase
LOX	lipoxygenase
*p*-TsOH	*p*-toluenesulfonic acid
MeCN	acetonitrile
EDG	Eriodictyol 7-O-glucuronide
RG	Reference Gene
SE	Standard Error
SD	Standard Deviation
