# Supplementary material for: JA-Ile-Macrolactone 5b Induces Tea Plant (Camellia sinensis) Resistance to Both Herbivore Ectropis obliqua and Pathogen Colletotrichum camelliae
Source: Int J Mol Sci. 2020 Mar 6;21(5):1828. doi: 10.3390/ijms21051828 (PMC7084730; doi:10.3390/ijms21051828)
Supplement: Supplementary file 1 [file ijms-21-01828-s001.pdf]

Supplementary Materials:

JA-Ile-macrolactone 5b induces tea plant (*Camellia sinensis*) resistance to both herbivore *Ectropis obliqua* and pathogen *Colletotrichum camelliae*

Songbo Lin [1,2], Yanan Dong [1,2], Xiwang Li [1,2], Yuxian Xing [1,2], Miaomiao Liu [1,2] and Xiaoling Sun [1,2,*]

Supplementary Methods

NMR data of JA-Ile-macrolactones. JA-Ile-macrolactone 5a. ^{1}H NMR (400 MHz, $CDCl_3$) δ 5.76 (d, *J* = 8.8 Hz, 1H), 5.35 (dddt, *J* = 10.4, 7.7, 5.6, 2.1 Hz, 1H), 5.10 (dt, *J* = 11.7, 6.3 Hz, 1H), 4.49 (ddd, *J* = 10.8, 4.4, 3.1 Hz, 1H), 4.41 (dd, *J* = 8.8, 6.7 Hz, 1H), 3.83 (td, *J* = 11.0, 1.9 Hz, 1H), 2.64 – 2.51 (m, 1H), 2.51 – 2.30 (m, 4H), 2.29 – 1.95 (m, 7H), 1.84 (dtd, *J* = 9.3, 6.8, 3.9 Hz, 1H), 1.58 (qd, *J* = 11.5, 9.3 Hz, 1H), 1.41 (dqd, *J* = 15.0, 7.5, 4.0 Hz, 1H), 1.08 (ddt, *J* = 14.4, 9.2, 7.3 Hz, 1H), 0.90 – 0.81 (m, 6H). ^{13}C NMR (100 MHz, $CDCl_3$) δ 220.30, 171.04, 170.69, 128.82, 128.37, 77.37, 77.26, 77.05, 76.73, 63.32, 57.30, 53.38, 42.98, 38.33, 37.50, 36.43, 28.59, 27.82, 25.60, 25.04, 15.73, 11.31.

JA-Ile-macrolactone 5b. ^{1}H NMR (400 MHz, $CDCl_3$) δ 5.68 (d, *J* = 8.1 Hz, 1H), 5.42 – 5.32 (m, 2H), 4.41 – 4.29 (m, 2H), 3.88 (ddd, *J* = 10.9, 6.3, 2.8 Hz, 1H), 2.59 – 2.47 (m, 2H), 2.42 (dtd, *J* = 14.6, 8.5, 2.6 Hz, 1H), 2.36 – 2.24 (m, 3H), 2.24 – 2.10 (m, 3H), 2.07 – 1.99 (m, 1H), 1.93 (dd, *J* = 5.9, 3.8 Hz, 2H), 1.63 – 1.46 (m, 1H), 1.39 (dtd, *J* = 14.8, 7.4, 4.3 Hz, 1H), 1.25 – 1.16 (m, 1H), 1.12 (ddd, *J* = 13.7, 9.3, 7.2 Hz, 1H), 0.94 – 0.80 (m, 6H). ^{13}C NMR (100 MHz, $CDCl_3$) δ 219.84, 171.53, 171.05, 129.24, 127.77, 77.36, 77.25, 77.04, 76.73, 64.21, 57.64, 55.82, 42.64, 37.78, 37.27, 36.10, 28.10, 27.04, 25.17, 25.10, 15.94, 11.48.

Supplementary Legends:

Table S1. Primers used in this investigation.

Table S2. MRM transitions and retention times of 12 flavonoids

Figure S1. Calibration curves of 12 flavonoids

Figure S2. ^{1} H and ^{13}C NMR Spectra of compounds

Table S1. Primers used in this investigation.

Name	GeneBank Accession Number	Primer Sequence (5′–3′) Forward/Reverse	Ref.
CsEF1	KA280301.1	TTGGACAAGCTCAAGGCTGAACG	[1]
		ATGGCCAGGAGCATCAATGACAGT	
CsCLATHRIN1	KA291473.1	TAGAGCGGGTAGTGGAGACCTCGTT	[1]
		TACCAAAGCCGGCTCGTATGAGATT	
CsACTIN1	KA280216.1	TGGGCCAGAAAGATGCTTATGTAGG	[2]
		ATGCCAGATCTTTTCCATGTCATCC	
CsGAPDH1	KA295375.1	TTTTTGGCCTTAGGAACCCAGAGG	[3]
		GGGCAGCAGCCTTATCCTTATCAGT	
CsSAND1	KM057790	TCCAATTGCCCCCTTAATGACTCA	[1]
		GTAAGGGCAGGCAAACACCAGGTA	
CsTIP41	AT4G34270	TGGAGTTGGAAGTGGACGAGACCGA	[4]
		CTCTGGAAAGTGGGATGTTTGAAGC	
CsUBC1	KA281185.1	TGCTGGTGGGGTTTTTCTTGTTACC	[1]
		AAGGCATATGCTCCCATTGCTGTTT	
CsPTB1	GAAC01052498.1	TGACCAAGCACACTCCACACTATCG	[1]
		TGCCCCCTTATCATCATCCACAA	
CsTUA1	JN399223.1	TCACTGTTTACCCATCTCCC	[3]
		GTAGGTGGGTCGCTCAATAT	
CsTBP	AT1G55520	GGCGGATCAAGTGTTGGAAGGGAG	[4]
		ACGCTTGGGATTGTATTCGGCATTA	
CsOPR3	XM_028243785.1	CGATCAACAGCCGGTGGATTT	[5]
		GCGTGGACAGCATCAACCAC	

Table S2. MRM transitions and retention times of 12 flavonoids.

Flavonoid Name	Transitions (*m*/*z*)	Retention Time (min)
Naringenin	273>153	9.37
Apigenin-5-o-glucoside	433>271	6.23
Cosnosiin	433>271	7.22
Isovitexin	433>283	5.73
Prunin	435>273	7.25
Astiagalin	449>287	6.95
Homoorientin	449>298	4.79
Eriodictuol-7-o-glucoside	451>289	5.99
Isoquercitrin	465>303	6.03
Neoschaftoside	565>271	6.87
Carlinoside	581>273	6.63
Rutin	611>303	5.60

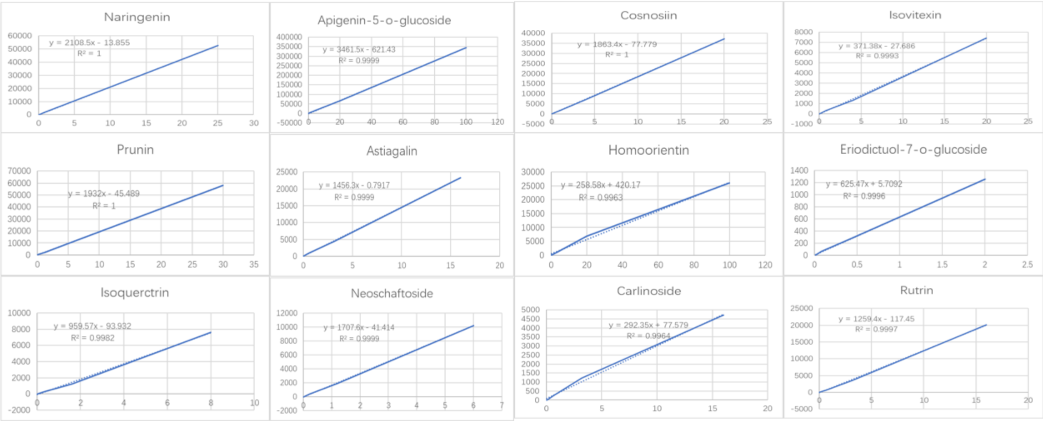

Figure S1. Calibration curves of 12 flavonoids.

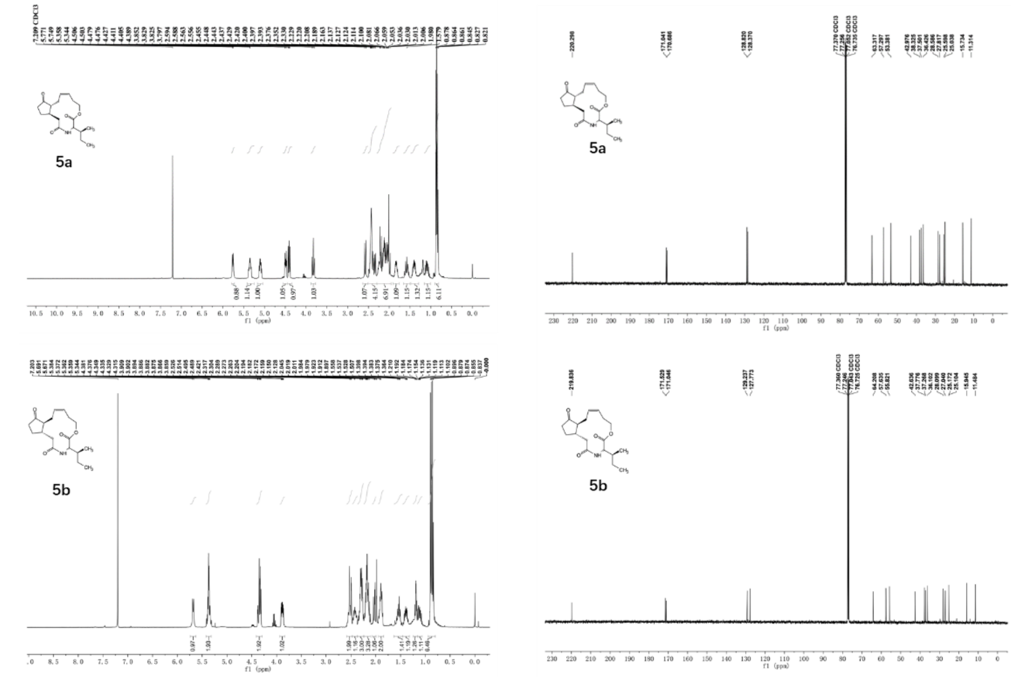

Figure S2. ^{1}H and ^{13}C NMR Spectra of compounds.

References

1. Hao, X.Y.; Horvath, D.P.; Chao, W.S.; Yang, Y.J.; Wang, X.C.; Xiao, B. Identification and Evaluation of Reliable Reference Genes for Quantitative Real-Time PCR Analysis in Tea Plant (Camellia sinensis (L.) O. Kuntze). *Int. J. Mol. Sci.* **2014,** *15*, 22155–22172.
2. Wang, M.L.; Li, Q.H.; Xin, H.H.; Chen, X.; Zhu, X.J.; Li, X.H. Reliable reference genes for normalization of gene expression data in tea plants (Camellia sinensis) exposed to metal stresses. *Plos ONE* **2017,** *12*, e0175863.
3. Ma, Q.P.; Hao, S.; Chen, X.; Li, X.H. Validation of reliability for reference genes under various abiotic stresses in tea plant. *Russ. J. Plant Physl.* **2016,** *63*, 423–432.
4. Wu, Z.J.; Tian, C.; Jiang, Q.; Li, X.H.; Zhuang, J. Selection of suitable reference genes for qRT-PCR normalization during leaf development and hormonal stimuli in tea plant (Camellia sinensis). *Sci. Rep.* **2016,** *6*, 19748.
5. Xin, Z.J.; Zhang, J.; Ge, L.G.; Lei, S.; Han, J.J.; Zhang, X.; Li, X. W.; Sun, X.L., A putative 12-oxophytodienoate reductase gene CsOPR3 from Camellia sinensis, is involved in wound and herbivore infestation responses. *Gene* **2017,** *615*, 18–24.
